# Impact of an outpatient palliative care consultation and symptom clusters in terminal patients at a tertiary care center in Pakistan

**DOI:** 10.1186/s12904-023-01195-4

**Published:** 2023-06-21

**Authors:** Wardah Rafaqat, Abbas Raza Syed, Ibrahim Munaf Ahmed, Shiraz Hashmi, Ismat Jabeen, Samina Rajwani, Uqba Qamar, Muhammad Atif Waqar

**Affiliations:** 1 Medical College, Aga Khan University Hospital, Karachi, Pakistan; 2 Senior Instructor, Department of Surgery, Aga Khan University Hospital, Karachi, Pakistan; 3 Section of Palliative Medicine, Department of Oncology, Aga Khan University Hospital, Karachi, Pakistan

**Keywords:** Palliative care, Symptom clusters, Symptom assessment, End-of-life care, Outpatient consultation

## Abstract

**Background:**

Patients with terminal diseases may benefit physically and psychosocially from an outpatient palliative care visit. Palliative care services are limited in Pakistan. An improved understanding of the symptom clusters present in our population is needed. The first outpatient palliative care center in Karachi, Pakistan, was established at our tertiary care institution. The primary aim of this study was to evaluate the impact of a palliative care outpatient consultation on symptom burden in patients with a terminal diagnosis. The secondary aim was to analyze the symptom clusters present in our population.

**Methods:**

Patients with a terminal diagnosis referred to our outpatient palliative department between August 2020-August 2022 were enrolled. The Edmonton Symptom Assessment Scale (ESAS) questionnaire was administered at the initial visit and the first follow-up visit at one month. Change in symptom burden was assessed using a Wilcoxon signed ranks test. A principal component analysis with varimax rotation was performed on the symptoms reported at the initial visit to evaluate symptom clusters. The palliative performance scale (PPS) was used to measure the performance status of palliative care patients.

**Results:**

Among the 78 patients included in this study, the average age was 59 ± 16.6 years, 52.6% were males, 99% patients had an oncological diagnosis, and the median duration between two visits was 14 (Q1-Q3: (7.0, 21.0) days. The median PPS level was 60% (Q1-Q3: 50–70). Overall, ESAS scores decreased between the two visits (6.0 (2.8, 11.0), *p* < 0.001) with statistically significant improvement in pain (5.0 vs. 2.5, *p* < 0.001), loss of appetite (5.0 vs. 4.0, *p* = 0.004), depression (2.0 vs. 0.0, *p* < 0.001), and anxiety (1.5 vs. 0.0, *p* = 0.032). Based on symptoms at the initial visit, 3 clusters were present in our population. Cluster 1 included anxiety, depression, and wellbeing; cluster 2 included nausea, loss of appetite, tiredness, and shortness of breath; and cluster 3 included drowsiness.

**Conclusion:**

An outpatient palliative care visit significantly improved symptom burden in patients with a terminal diagnosis. Patients may benefit from further development of outpatient palliative care facilities to improve the quality of life in terminally ill patients.

## Introduction

Patients with terminal diseases may experience a combination of physical symptoms like exhaustion due to the condition along with psychosocial symptoms like anxiety and sadness [1]. Previous studies have shown that multiple concurrent symptoms are common in patients with terminal illnesses, particularly severe conditions like metastatic cancer. Two or more linked symptoms that appear concurrently are referred to as a symptom cluster [2, 3]. Prior studies have reported clusters between dyspnea, fatigue, and depression [2]; insomnia, anorexia, weight loss, and tiredness; and nausea and vomiting [4]. Symptom clusters are significant because they may have similar etiology and can be treated according to inclusive symptom management models [5].

Palliative care tries to alleviate the physical and psychosocial suffering of terminally ill patients by augmenting medical interventions and recognizing and optimizing the management of symptom clusters [6, 7]. Additionally, it provides caregivers assistance to help them accept the patient's sickness and establish plans to care for them during treatment [8].

Formal palliative care services are limited in the public and private healthcare sectors in Pakistan [9]. Our institution is the first in Karachi to establish an outpatient palliative care program. Outpatient palliative care provides continuity of care to a broader range of patients than inpatient facilities. Recent literature has shown an improvement in symptom burden when patients with terminal diagnoses avail outpatient palliative care services [10, 11].

However, the impact of outpatient palliative care consultations on our population remains to be seen. There is limited research regarding symptom clusters present in palliative care patients in South Asia. A greater understanding of the symptom clusters present in this population may result in better symptom control and an improvement in quality of life [5].

Therefore, the primary aim of this study was to evaluate the impact of a palliative care outpatient consultation at a tertiary care center, The secondary aim was to analyze the symptom clusters present in our population.

## Methods

### Patient population

This prospective observational study examines the minimal clinically significant difference between the Edmonton Symptom Assessment Scale (ESAS) at the patient’s initial visit and the first follow-up visit 7–21 days after the initial visit. Patients aged 18 years and above receiving their first palliative appointment at our institution’s outpatient palliative care clinic (initial visit) at any stage of their disease between August 2020 to August 2022 were included. Patients who were unable to follow up within 21 days of their first appointment and those who refused to participate were excluded from the study. The study was approved by the Ethics Review Committee (2020–3439-11,093). Informed consent was taken from every patient to participate in the study. All methods were performed in accordance with the relevant guidelines and regulations.

### Process of assessment

Palliative patients at our institution receive treatment from a comprehensive team consisting of 3 specialists who provide coverage in cases of absence or illness to ensure a homogenous approach. The team also consists of registered nurses trained especially for palliative care. For any specialized needs that may arise, referrals are given to specialists in other fields for a multi-disciplinary, holistic approach.

At the outpatient clinic, the patient is assessed by the consultant, who reviews the history and physically examines the patient. Then the consultant proceeds to counsel the family and the patient, together or separately, if the need arises. The study included patients attending an in-person office visit or a telehealth audio or video consultation. For the inpatient visit, the patient was provided English or Urdu consent form in their preferred language and a separate room for privacy. The English or Urdu consent form was given to the patient according to their preference. A study staff was available to answer any questions that may have arisen during this process. Once consent was received, the patient was provided with the ESAS questionnaire and requested to fill it out. Patients with visual impairment who were unable to read the form or physical limitations due to which they were unable to fill out the form were given the option of receiving assistance from their attendant or a study staff member. Assistance was limited to reading out the form to the patient and marking the response provided by the patient on the form. This ensured that bias was minimized and the patient was able to record their responses. At the follow-up visit, a similar protocol was followed when the patient was provided with the ESAS questionnaire to mark the recent burden of their symptoms.

For telehealth visits, the study was explained, and the consent form was read out to the patient during the initial tele-appointment by a study staff member, and any questions raised by the patient were addressed. Once verbal consent was recorded, the ESAS questionnaire was read out to the patient and the responses to the questionnaire were recorded by a study staff member. Similarly, a study staff member read out the questionnaire to the patient at the follow-up visit, and recorded the patient’s responses.

### Study instruments

The ESAS was initially developed by Bruera et. al. [12] as a clinical tool to assess the severity of symptoms in patients with advanced cancer. Since then, it has been validated by several studies [13, 14] and its scope has been increased to include the burden of disease in all palliative patients [12]. ESAS can be used to assess the symptom severity at a point of time and can be used to monitor in interval follow ups as well, therefore showing the impact of different treatment modalities.

The nine ESAS questions' scores are added up to produce the symptom distress score. A numerical rating scale (0–10) for several symptoms, such as pain, fatigue, nausea, depression, anxiety, drowsiness, appetite, wellbeing, and shortness of breath, is included in the ESAS-R questionnaire. The patients were asked to rate the symptoms according to the severity experienced in the past 24 h, with higher numbers indicating greater symptom intensity [14]. The final score of the patient was on a continuous scale.

The English ESAS questionnaire was translated into Urdu, which is the national language of Pakistan, according to the EORTC protocol [15]. Two native Urdu speakers with fluency in the English language initially translated the questionnaire into Urdu. The differences were reconciled with assistance from a third professional translator. The reconciled translation was translated back into English by two native Urdu speakers with fluency in English. They were not previously exposed to the English version of the questionnaire. These back translations were reviewed by the original translators and the principal investigator and a translated version was agreed upon. To ensure face validity, the Urdu version of the survey underwent pilot testing amongst 15 respondents. Ambiguity was not reported by the respondents so no further modifications were made.

The Palliative Performance Scale (PPS) has been used and validated in several countries and has been translated into other languages [16]. It is an adapted version of the Karnofsky scale [17]. PPS is scored via observation on a scale of 0% to 100% in 10% intervals. It includes five domains – Ambulation, Self-care, Activity Level/Evidence of Disease, Intake, and Level of Consciousness. PPS was used as a tool to measure the performance status of palliative care patients.

### Statistical analysis

Data were analyzed in STATA version 14.2. Descriptive statistics were used to summarize our data, including mean (± standard deviation) or median (IQR) for continuous variables and frequency and percentage tables for categorical data.

### Improvement of symptoms

Studies on the responsiveness of ESAS have shown that a change in one score for all ten physical symptoms is clinically significant [18]. The medians (IQR) of the total score and individual symptoms were calculated for the initial visit and the follow-up visits. A Wilcoxon sign ranked test was performed to compare the two phasic scores with the baseline. A *p*-value of < 0.05 was considered as significant.

### Symptom clusters

To determine the interrelationships between the 9 ESAS items, a principal component analysis with varimax rotation was performed on the symptoms reported at the patient’s first clinic visit. Correlation between the items, test of sphericity and sampling adequacy was determined. To determine the significant principal components, each of which accounted for at least 12% of the total variance, the highest eigenvalues (greater than 1.0) were utilized. Additionally, the final communality, the proportion of the variance in an observed variable that is explained by the retained components, was provided. To demonstrate strong correlations between the symptoms, a biplot graphic was developed. Arrows that were longer and closer together were thought to demonstrate a stronger association between symptoms.

## Results

### Patient demographics

A total of 103 patients were initially recruited in this study. However, 25 patients were unable to followup due to inpatient hospitalization or death before the scheduled follow-up appointment. Eventually, 78 patients were included in the study. The mean age of the population was 59 ± 16.6 years, out of which 41 (52.6%) were males. The most common diseases were cancers of organs in the abdominal cavity (liver, gallbladder, and pancreas) 28 (35.8%), head and neck cancers 11 (14.1%) followed by breast cancer 9 (11.5%) and others 15 (19.2%). The median time between the two visits was 14 days (IQR: 7–21 days). Out of 78 patients, 31 underwent concomitant treatment in the form of chemotherapy 13 (16.7%), radiotherapy, or surgery each 9 (11.5%). Median PPS level was 60% IQR:(50–70%), (Table 1).

**Table 1:** Demographics characteristics of study population, *n* = 78

Patient characteristics	Value (total *n* = 78)
Age Mean ± (SD)	59.0 ± 16.6
**Gender**	
- Male	41 (52.6%)
- Female	37 (47.4%)
**Primary cancer site / Diagnosis**	
- Abdominal cancers^a^	28 (35.8%)
- Head and neck cancers	11 (14.1%)
- Breast cancer	09 (11.5%)
- Gynecological cancers	07 (8.9%)
- CNS and neuroendocrine cancers	04 (5.1%)
- Renal cell carcinoma	04 (5.1%)
- Others^b^	15 (19.2%)
**Comorbidities**	
- Hypertension	6 (7.7%)
- Diabetes	4 (5.1%)
- Respiratory	1 (1.3%)
- Endocrine disorders	2 (2.6%)
- Hematological	2 (2.6%)
- Gastrointestinal problems	1 (1.3%)
**Currently on any treatment**	
- Chemotherapy	13 (16.7%)
- Radiotherapy	9 (11.5%)
- Surgery	9 (11.5%)
**PPS Score (%)**	
PPS level (Mean % ± SD)	59.0 (± 15.0)
PPS level [Median % (IQR)]	60.0 (50.0, 70.0)
**Form filled by**	
- Patient	69 (88.5)
- HCP	4 (5.1)
- Family member	5 (6.4)
Median follow up time^a^ (IQR) days	14.0 (7.0, 21.0)

^a^Includes cancers of GI Tract along with liver, gallbladder, pancreas

^b^Includes lung, bone, prostatic, retroperitoneal and unspecified cancers, leukemia and end-stage heart disease

### ESAS score comparison at initial and followup visit

**Fig. 1:**
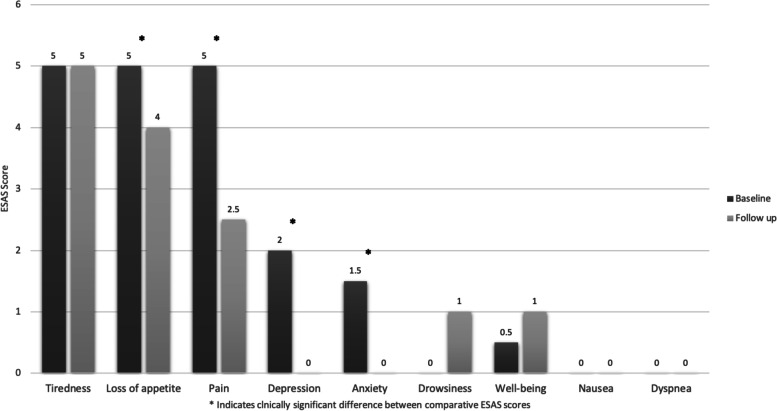
Comparison of Edmonton Symptom assessment scores at the initial and follow-up visit

There was an evident reduction in symptom ESAS scores between the first and second visits (Table 2 and Fig. 1). Pain (5 vs. 2.5, *P* < 0.001), loss of appetite (5 vs. 4, *p* = 0.004), depression (2 vs. 0, *p* < 0.001) and anxiety (1.5 vs. 0, *p* = 0.032) were all found to improve significantly after the initial outpatient visit. There was a significant reduction in the total median ESAS score at first follow up visit compared to the score at baseline initial visit 20 (IQR: 12.8–27.0) vs. 25.5 (IQR: 16.0–35.0), *p* < 0.001 (Table 3).

**Table 2:** Change in ESAS items score median (IQR) from baseline to follow up

Symptoms	Baseline Score	Follow up Score	*P* value*
- Pain	5.0 (1.0, 8.0)	2.5 (0.0, 5.0)	< 0.001
- Tiredness	5.0 (2.8, 8.0)	5.0 (2.0, 7.3)	0.187
- Drowsiness	0.0 (0.0, 5.0)	1.0 (0.0, 4.0)	0.574
- Nausea	0.0 (0.0, 1.0)	0.0 (0.0, 0.0)	0.239
- Loss of appetite	5.0 (1.0, 8.0)	4.0 (0.0, 6.0)	0.004
- Dyspnea	0.0 (0.0, 2.0)	0.0 (0.0, 1.3)	0.156
- Depression	2.0 (0.0, 5.0)	0.0 (0.0, 3.0)	< 0.001
- Anxiety	1.5 (0.0, 3.0)	0.0 (0.0, 3.0)	0.032
- Well-being	0.5 (0.0, 5.0)	1.0 (0.0, 4.0)	0.171
- Total ESAS Score	25.5 (16.0, 35.0)	20 (12.8, 27.0)	< 0.001

^*^Wilcoxon signed ranks test

**Table 3:** Eigenvalues and proportions of variance for PCA

Component	Eigen value	Total variance explained (%)	Cumulative
1^a^	2.724	30.271	30.271
2^a^	1.515	16.828	47.099
3^a^	1.065	11.832	58.931
4^a^	1.034	11.489	70.420
5	.853	9.483	79.903
6	.668	7.423	87.327
7	.575	6.392	93.718
8	.377	4.192	97.910

^a^Value represents the components with Eigenvalues > 1.0

### Symptom clusters

**Fig. 2:**
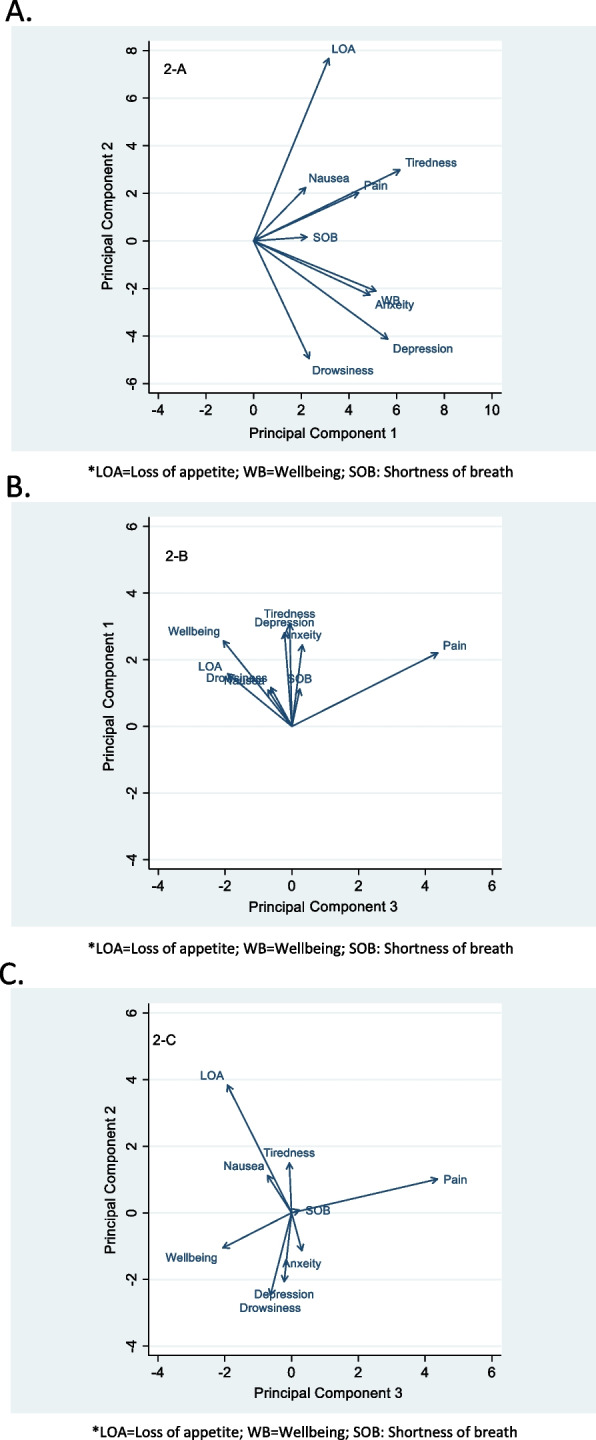
**A**-**C** Biplot graphs showing the correlation between symptoms according to the three components. The length of the lines correspond to the magnitude of factor loading values. Factor loading values indicate the contribution of each variable to the component. A high value indicates that the factor strongly influences the component. The angles between the lines correspond to their correlation; a small angle denotes a higher positive correlation, a right angle denotes no likely correlation, and large diverging angles (close to 180) show a negative correlation

Bartlett's test of sphericity was < 0.001, and the Kaiser–Meyer–Olkin Measure of Sampling Adequacy was 0.6. Three components with the highest eigenvalues were selected and accounted for more than 59% of the total variance (Tables 3 and 4). Components 1, 2, and 3 accounted for 30%, 17%, and 12% of the total variance, respectively. Component 1 included anxiety, depression, and wellbeing, component 2 included nausea, loss of appetite, tiredness, and shortness of breath, component 3 included drowsiness (Fig. 2a-c). The Cronbach’s alpha value indicating internal consistency was 0.74 for the first cluster and 0.59 for the second cluster. For component 3, only one item of drowsiness qualified, so alpha could not be assessed. The final commonality determined that all components were accounted for within the 3 clusters, with final estimates ranging from 0.35 for shortness of breath to 0.80 for depression (Tables 3 and 4).

**Table 4:** Factor loadings and final communality for PCA

Symptom	Components			Final communality
	**1**	**2**	**3**	
Anxiety^a^	0.5663^a^	-0.0493	-0.0037	0.737
Depression^a^	0.5521^a^	-0.0803	0.2039	0.800
Wellbeing^a^	0.4020^a^	-0.0068	-0.0115	0.380
Nausea	-0.1238	0.6315^a^	0.2487	0.667
Loss of appetite	-0.0378	0.5316^a^	-0.3538	0.653
Tiredness	0.2889	0.4173^a^	-0.1642	0.650
SOB	0.0856	0.3591^a^	0.2572	0.350
Drowsiness	0.0861	0.0618	0.7201^a^	0.680
Pain	0.3131	0.0532	-0.3994	0.387
Percent (%) of variance	0.3027	0.1683	0.1183	-
Cronbach’s alpha	0.7473	0.5912	-	-

^a^Values represent distinct clusters related to factor loading scores. Factor loading values indicate the contribution of each variable to the component. A high value indicates that the factor strongly influences the component

Figure 2a-c are biplot graphs showing the correlation between symptoms according to the three components. The length of the lines corresponds to the magnitude of factor loading values. Factor loading values indicate the contribution of each variable to the component. A high value indicates that the factor strongly influences the component. The angles between the lines correspond to their correlation; a small angle denotes a higher positive correlation, a right angle denotes no likely correlation, and large diverging angles (close to 180) show a negative correlation.

## Discussion

This study is the first in our knowledge to assess the impact of outpatient palliative care visit on symptom burden and the presence of symptom clusters in our population. Seven out of nine components of the ESAS scale were prevalent among our patients. There was a marked reduction in total symptom load for patients who visited the outpatient palliative medicine clinic. The symptom burden of pain, loss of appetite, depression, and anxiety showed statistically significant improvement (Fig. 1).

Palliative care is still relatively new to national health systems, particularly in low-and middle-income countries (LMICs) [19]. Pakistan was classified as category 3a (Isolated palliative care provision) in 2017 by a global study that tracks the evolution of palliative care services to classify nations according to degrees of palliative care development [20]. There are very few medical facilities in Pakistan that provide palliative care facilities [21]. In the private sector, palliative care is available in Shaukat Khanum Memorial Cancer Hospital, Lahore, Aga Khan University Hospital (AKUH), Karachi, Children’s Cancer Hospital, Karachi [9], and a few Christian hospices in Karachi, Hyderabad and Rawalpindi. AKUH is the only hospital in Karachi that offers outpatient palliative care services. The outpatient palliative care consultation was associated with improved scores in referred patients. Previous studies have shown significant improvements in several ESAS domains (pain, fatigue, drowsiness, nausea, loss of appetite, dyspnea, depression, anxiety, and wellbeing) following outpatient palliative care consultation [10, 11]. A similar decrease in symptom load was also seen in our study.

The median scores for nausea, dyspnea, worst wellbeing, drowsiness, anxiety, and depression reported at baseline (initial visit) as reported by patients in the ESAS questionnaire were lower in our population compared to previous studies [22, 23]. For example, one of the questions included within the ESAS questionnaire was regarding the patient’s perception of their wellbeing, with values closer to 0 indicating the best wellbeing. The median score in our population was 0.5 and 1 before and after the consultation, respectively. In recent literature, the “wellbeing” score assessed using the ESAS questionnaire ranged between 3 to 6, which was higher than our population [22, 24, 25]. Several factors could be responsible for lower scores, including the oncological department physicians’ capability to treat these symptoms before referral and the type of malignancy present in the patient.

We found a significant decrease in pain, loss of appetite, depression, and anxiety after the palliative care consultation. Previous studies have reported similar benefits of an outpatient consultation. Kang et al., reported a significant decrease in the burden of fatigue, pain, nausea, depression, anxiety, drowsiness, dyspnea, loss of appetite, sleep disturbances, and improved wellbeing after a palliative care consultation for patients with advanced cancer [22].

In another study, Yennurajalingam et al. found significant improvements in pain, drowsiness, fatigue, depression, sleep, sense of wellbeing, and anxiety at the first follow-up visit among prostate cancer patients referred to palliative care [26].

While the clusters present in previous studies are highly variable due to the difference in instruments used to measure the symptom variables or the methods used to analyze the symptom clusters, a systematic review based on 33 articles found four common groupings, being anxiety-depression, nausea-vomiting, nausea-appetite loss, and fatigue-dyspnea-drowsiness-pain [27]. In our study, anxiety and depression were present in one cluster, and nausea and loss of appetite were also present in one cluster. It was interesting to note that pain was not present in the common groupings or the clusters in our population. Pain was a prevalent symptom in our population, but previous studies have hypothesized that an underlying mechanism may cause clusters to form. The mechanism behind pain may not align with that of other symptoms consistently enough for it to form a cluster. It has also been hypothesized that individual susceptibilities may drive a combination of symptoms within patients, which then cumulatively form a symptom cluster and make it difficult to predict symptom clusters within a population [28]. Understanding symptom clusters can enable a thorough symptom evaluation and management by allowing the physician to anticipate symptoms [22].

Multiple studies suggest that PPS is a significant predictor of survival for patients with both cancer and other end-of-life diagnoses [16, 17]. According to the literature, early referral to palliative care, at least 3 months before death, should be a standard of care in oncological practice [29]. It has been shown to reduce hospital length of stay and intensive care unit hospitalization [30]. Recent literature suggests that a PPS score of 60 percent generally indicates a median survival of 35 to 43 days [10, 31, 32]. The median PPS score in our population was 60% at the time of referral indicating that timely patient referral was not occurring in our population.

The symptom clusters found in our patient population are consistent with those reported in other studies [25, 33, 34]. In our study, the cluster containing symptoms of anxiety, depression, and wellbeing is in accordance with other studies, which also found a psychoneurological symptom cluster among other clusters [34]. However, the types of symptom clusters and their exact components identified in past literature have been highly variable. This may be due to the difference in instruments used to measure the symptom variables or the methods used to analyze the symptom clusters [18]. Understanding symptom clusters enables a more thorough symptom evaluation since knowing symptoms that present together can facilitate the physician in anticipating other symptoms and treating them accordingly. Moreover, being cognizant of the co-occurrence of particular symptoms opens up the possibility of more effective symptom management by focusing on the cluster of symptoms with a single treatment strategy [29].

### Limitations

One of the limitations of this study is that there may be confounding variables that impact the symptoms experienced by the patient, including the progression of the patient's disease or concurrent disease-modifying therapy. To reduce the impact of this limitation, the authors restricted the follow-up period to 21 days. The rate of attrition in our study was 76%. The loss of patients to follow-up may have contributed to attrition bias in our study. However, this rate is similar to other studies assessing the impact of an outpatient consultation on symptoms in palliative care patients [35].

The ESAS questionnaire offers limited granularity about the symptoms included and remains limited to ten symptoms. Augmenting the ESAS questionnaire with validated symptom assessment scales such as the Memorial symptom assessment scale may have offered a more holistic understanding [36]. Moreover, the ESAS questionnaire captures the recent severity of the patient’s symptoms. Given the fluctuating nature of the metastatic disease, the symptom burden reported by the patient may not be fully representative of the severity of their symptoms [37, 38]. The patient population in our study comprises a majority of oncological patients so this study’s findings may have limited applicability to terminally ill patients with non-metastatic disease. Finally, we used only one method of data analysis to form symptom clusters. Other methods, such as exploratory factor analysis and hierarchal cluster analysis, may have identified other clusters that were not captured by PCA [33].

## Conclusion

An outpatient palliative care consultation at our institution was associated with improved pain, loss of appetite, depression, and anxiety. Symptom clusters between anxiety, depression, wellbeing and nausea, loss of appetite, shortness of breath, and tiredness were found. Terminally ill patients may benefit from further development of outpatient palliative care facilities to improve their quality of life.

## Data Availability

The datasets used and/or analyzed during the current study are available from the corresponding author on reasonable request.

## References

[1] Harrison JD, Young JM, Price MA, Butow PN, Solomon MJ (2009). What are the unmet supportive care needs of people with cancer? A systematic review. Support Care Cancer Off J Multinatl Assoc Support Care Cancer.

[2] Chen E, Nguyen J, Cramarossa G, Khan L, Zhang L, Tsao M (2012). Symptom clusters in patients with advanced cancer: sub-analysis of patients reporting exclusively non-zero ESAS scores. Palliat Med.

[3] Dodd MJ, Miaskowski C, Paul SM (2001). Symptom clusters and their effect on the functional status of patients with cancer. Oncol Nurs Forum.

[4] Jiménez A, Madero R, Alonso A, Martínez-Marín V, Vilches Y, Martínez B (2011). Symptom clusters in advanced cancer. J Pain Symptom Manage.

[5] Xiao C (2010). The state of science in the study of cancer symptom clusters. Eur J Oncol Nurs Off J Eur Oncol Nurs Soc.

[6] Levy MH, Back A, Benedetti C, Billings JA, Block S, Boston B (2009). NCCN clinical practice guidelines in oncology: palliative care. J Natl Compr Cancer Netw JNCCN.

[7] Ferris FD, Bruera E, Cherny N, Cummings C, Currow D, Dudgeon D (2009). Palliative cancer care a decade later: accomplishments, the need, next steps – from the American Society of Clinical Oncology. J Clin Oncol Off J Am Soc Clin Oncol.

[8] WHO (2020) Palliative care. In: World Health Organization. https://www.who.int/news-room/fact-sheets/detail/palliative-care. Accessed 21 June 2023.

[9] Abbas SQ, Abbas SZ (2002). Palliative medicine: an emerging discipline. JPMA J Pak Med Assoc.

[10] Shah R, Georgousopoulou EN, Al-Rubaie Z, Sulistio M, Tee H, Melia A (2022). Impact of ambulatory palliative care on symptoms and service outcomes in cancer patients: a retrospective cohort study. BMC Palliat Care.

[11] Shamieh O, Khamash O, Khraisat M, Jbouri O, Awni M, Al-Hawamdeh A (2017). Impact of outpatient palliative care (PC) on symptom burden in patients with advanced cancer at a tertiary cancer center in Jordan. Support Care Cancer Off J Multinatl Assoc Support Care Cancer.

[12] Bruera E, Kuehn N, Miller MJ, Selmser P, Macmillan K (1991). The Edmonton Symptom Assessment System (ESAS): A Simple Method for the Assessment of Palliative Care Patients. J Palliat Care.

[13] Philip J, Smith WB, Craft P, Lickiss N (1998). Concurrent validity of the modified Edmonton Symptom Assessment System with the Rotterdam Symptom Checklist and the Brief Pain Inventory. Support Care Cancer Off J Multinatl Assoc Support Care Cancer.

[14] Yennu S, Urbauer DL, Bruera E (2012). Factors associated with the severity and improvement of fatigue in patients with advanced cancer presenting to an outpatient palliative care clinic. BMC Palliat Care.

[15] Dewolf L, Koller M, Velikova G, Johnson C, Scott N, Bottomley A et al. EORTC Quality of Life Group translation procedure. 3rd ed. Brussels, Belgium: EORTC Quality of Life Group. 2009. p. 32.

[16] Ho F, Lau F, Downing MG, Lesperance M (2008). A reliability and validity study of the Palliative Performance Scale. BMC Palliat Care.

[17] Baik D, Russell D, Jordan L, Dooley F, Bowles KH, Masterson Creber RM (2018). Using the palliative performance scale to estimate survival for patients at the end of life: A systematic review of the literature. J Palliat Med.

[18] Hui D, Bruera E (2017). The Edmonton Symptom Assessment System 25 Years Later: Past, Present, and Future Developments. J Pain Symptom Manage.

[19] Osta BE, Palmer JL, Paraskevopoulos T, Pei BL, Roberts LE, Poulter VA (2008). Interval between first palliative care consult and death in patients diagnosed with advanced cancer at a comprehensive cancer center. J Palliat Med.

[20] Clark D, Baur N, Clelland D, Garralda E, López-Fidalgo J, Connor S (2020). Mapping Levels of Palliative Care Development in 198 Countries: The Situation in 2017. J Pain Symptom Manage.

[21] Shad A, Ashraf MS, Hafeez H (2011). Development of palliative-care services in a developing country: Pakistan. J Pediatr Hematol Oncol.

[22] Kang JH, Kwon JH, Hui D, Yennurajalingam S, Bruera E (2013). Changes in symptom intensity among cancer patients receiving outpatient palliative care. J Pain Symptom Manage.

[23] Yennurajalingam S, Kwon JH, Urbauer DL, Hui D, Reyes-Gibby CC, Bruera E (2013). Consistency of symptom clusters among advanced cancer patients seen at an outpatient supportive care clinic in a tertiary cancer center. Palliat Support Care.

[24] Cervantez SR, Tenner LL, Schmidt S, Aduba IO, Jones JT, Ali N (2018). Symptom Burden and Palliative Referral Disparities in an Ambulatory South Texas Cancer Center. Front Oncol.

[25] Rauenzahn SL, Schmidt S, Aduba IO, Jones JT, Ali N, Tenner LL (2017). Integrating Palliative Care Services in Ambulatory Oncology: An Application of the Edmonton Symptom Assessment System. J Oncol Pract.

[26] Yennurajalingam S, Atkinson B, Masterson J, Hui D, Urbauer D, Tu SM (2012). The impact of an outpatient palliative care consultation on symptom burden in advanced prostate cancer patients. J Palliat Med.

[27] Dong ST, Butow PN, Costa DSJ, Lovell MR, Agar M (2014). Symptom clusters in patients with advanced cancer: a systematic review of observational studies. J Pain Symptom Manage.

[28] Miaskowski C, Barsevick A, Berger A, Casagrande R, Grady PA, Jacobsen P (2017). Advancing Symptom Science Through Symptom Cluster Research: Expert Panel Proceedings and Recommendations. JNCI J Natl Cancer Inst.

[29] Boltezar L, Novakovic BJ, Moltara ME (2021). Trends in specialized palliative care referrals at an oncology center from 2007 to 2019. BMC Palliat Care.

[30] Robbins SG, Hackstadt AJ, Martin S, Shinall MC (2019). Implications of Palliative Care Consultation Timing among a Cohort of Hospice Decedents. J Palliat Med.

[31] Weng LC, Huang HL, Wilkie DJ, Hoenig NA, Suarez ML, Marschke M (2009). Predicting survival with the Palliative Performance Scale in a minority-serving hospice and palliative care program. J Pain Symptom Manage.

[32] Yoon SJ, Choi SE, LeBlanc TW, Suh SY (2018). Palliative Performance Scale Score at 1 Week After Palliative Care Unit Admission is More Useful for Survival Prediction in Patients With Advanced Cancer in South Korea. Am J Hosp Palliat Care.

[33] Chen E, Nguyen J, Khan L, Zhang L, Cramarossa G, Tsao M (2012). Symptom clusters in patients with advanced cancer: a reanalysis comparing different statistical methods. J Pain Symptom Manage.

[34] Kim HJ, McDermott PA, Barsevick AM (2014). Comparison of groups with different patterns of symptom cluster intensity across the breast cancer treatment trajectory. Cancer Nurs.

[35] Preston NJ, Fayers P, Walters SJ, Pilling M, Grande GE, Short V (2013). Recommendations for managing missing data, attrition and response shift in palliative and end-of-life care research: part of the MORECare research method guidance on statistical issues. Palliat Med.

[36] Ingham JM, Portenoy RK (1996). Symptom assessment. Hematol Oncol Clin North Am.

[37] McClement SE, Woodgate RL, Degner L (1997). Symptom distress in adult patients with cancer. Cancer Nurs.

[38] Hayduk L, Olson K, Quan H, Cree M, Cui Y (2010). Temporal changes in the causal foundations of palliative care symptoms. Qual Life Res Int J Qual Life Asp Treat Care Rehabil.

